# Supplementary material for: MicroRNA-100 Mediates Hydrogen Peroxide-Induced Apoptosis of Human Retinal Pigment Epithelium ARPE-19 Cells
Source: Pharmaceuticals (Basel). 2021 Apr 1;14(4):314. doi: 10.3390/ph14040314 (PMC8067261; doi:10.3390/ph14040314)
Supplement: Supplementary file 1 [file pharmaceuticals-14-00314-s001.pdf]

Supplementary Figure

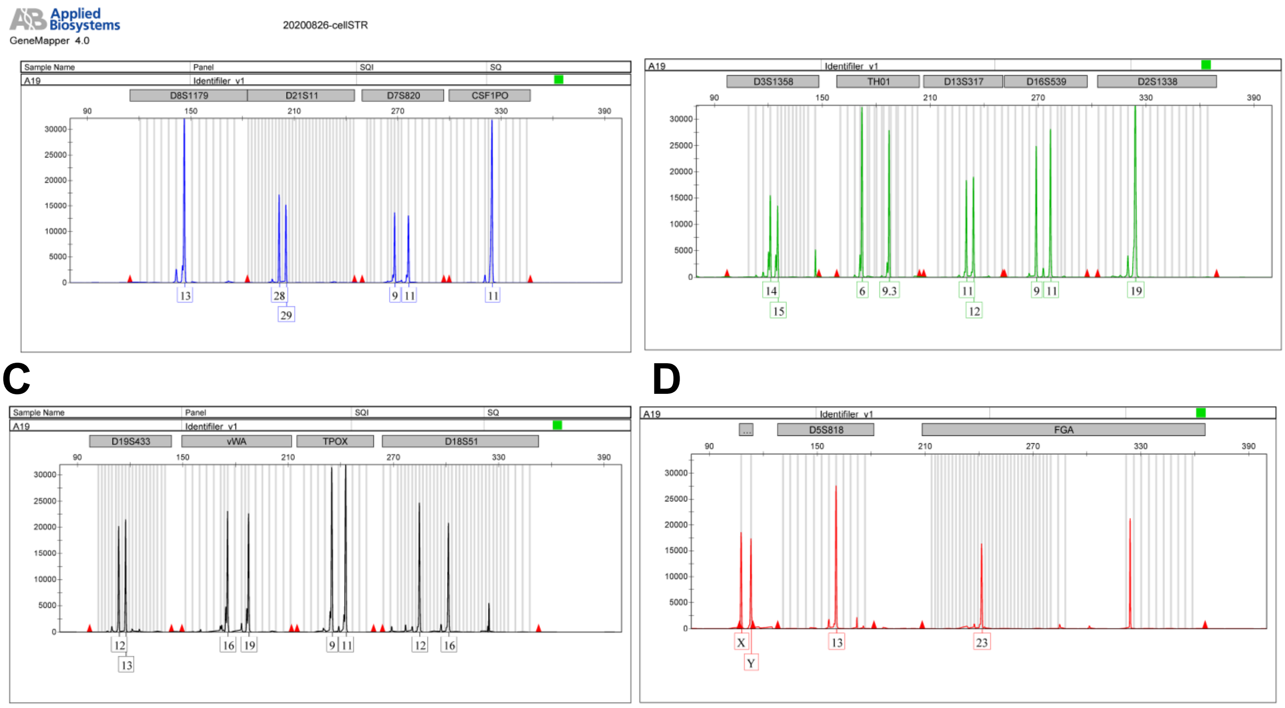

E

Result of STR matching analysis by your data.

- DSMZ Profile Database -

A graphical presentation is shown at the bottom of this page.

EV	Cell No.	Cell name	Locus names									Figures
			D5S818	D13S317	D7S820	D16S539	VWA	TH01	AM	TPOX	CSF1PO	
	Query (Your Cell)		*13,13*	*11,12*	*9,11*	*9,11*	*16,19*	*6,9.3*	*x,y*	*9,11*	*11,11*	
1.00(36/36)	CRL-2302	ARPE-19	13,13	11,12	9,11	9,11	16,19	6,9.3	X,Y	9,11	11,11	-
1.00(36/36)	CRL-2502	ARPE-19/HPV-16	13,13	11,12	9,11	9,11	16,19	6,9.3	X,Y	9,11	11,11	-

Supplementary Figure S1. Short tandem repeat (STR) genotyping analysis on ARPE-19 cell line used in the study. The genomic DNA of cultured ARPE-19 cells was extracted using DNeasy Blood & Tissue Kit (Qiagen#69504) and subjected to multiplex PCR amplification with AmpFLSTR^{TM} Identifiler^{TM} Plus Kit (ThermoFisher#4427368), which simultaneously detected 16 human gene loci. The STR PCR amplified products were resolved by a fluorescent capillary electrophoresis instrument (DNA Analyzer 3730XL), and analyzed by using GeneMapper v4.0 software. Panel (**A**) showing the detected gene loci and alleles in the tested cells, including D8S1179, D21S11, D7S820 and CSF1PO; panel (**B**), D3S1358, TH01, D13S317, D16S539, D2S1338; panel (**C**), D19S433, vWA, TPOX, D18S51; panel (**D**), D5S818 and FGA. (**E**) The data of STR matching analysis compared to ARPE-19 cell line (DSMZ no. CRL-2302) and ARPE-19/HPV-16 cell line (DSMZ no. CRL-2502) in DSMZ STR Profile Database showed that evaluation values (EV) of matching algorithm were both 1.0, which indentified that the cell line used in this study is ARPE-19.
